# Oleacein Prevents High Fat Diet-Induced Adiposity and Ameliorates Some Biochemical Parameters of Insulin Sensitivity in Mice

**DOI:** 10.3390/nu11081829

**Published:** 2019-08-07

**Authors:** Saverio Massimo Lepore, Valentina Maggisano, Stefania Bulotta, Chiara Mignogna, Biagio Arcidiacono, Antonio Procopio, Antonio Brunetti, Diego Russo, Marilena Celano

**Affiliations:** 1 Department of Health Sciences, University “Magna Graecia” of Catanzaro, 88100 Catanzaro, Italy; 2 Interdepartmental Service Center, University “Magna Graecia” of Catanzaro, 88100 Catanzaro, Italy

**Keywords:** oleacein, high-fat diet, obesity, abdominal fat, adipogenesis, Glut-4

## Abstract

Oleacein is one of the most abundant polyphenolic compounds of olive oil, which has been shown to play a protective role against several metabolic abnormalities, including dyslipidemia, insulin resistance, and glucose intolerance. Herein, we investigated the effects of oleacein on certain markers of adipogenesis and insulin-resistance in vitro, in 3T3-L1 adipocytes, and in vivo in high-fat diet (HFD)-fed mice. During the differentiation process of 3T3-L1 preadipocytes into adipocytes, oleacein strongly inhibited lipid accumulation, and decreased protein levels of peroxisome proliferator-activated receptor gamma (PPARγ) and fatty acid synthase (FAS), while increasing Adiponectin levels. In vivo, treatment with oleacein of C57BL/6JOlaHsd mice fed with HFD for 5 and 13 weeks prevented the increase in adipocyte size and reduced the inflammatory infiltration of macrophages and lymphocytes in adipose tissue. These effects were accompanied by changes in the expression of adipose tissue-specific regulatory elements such as PPARγ, FAS, sterol regulatory element-binding transcription factor-1 (SREBP-1), and Adiponectin, while the expression of insulin-sensitive muscle/fat glucose transporter Glut-4 was restored in HFD-fed mice treated with oleacein. Collectively, our findings indicate that protection against HFD-induced adiposity by oleacein in mice is mediated by the modulation of regulators of adipogenesis. Protection against HFD-induced obesity is effective in improving peripheral insulin sensitivity.

## 1. Introduction

Obesity-related insulin resistance (IR) is a dysmetabolic condition in which insulin target tissues (namely muscle, liver, and fat) fail to properly respond to insulin. From the clinical point of view, IR is a key feature of metabolic syndrome, and it can lead to the development of type 2 diabetes and cardiovascular disease [1,2,3]. As such, IR is a target for therapeutic interventions and can be reversed by dietary changes and nutraceuticals.

Olives and extra virgin olive oil (EVOO) are the major fat components of the Mediterranean diet [4]. Several studies in this context have suggested that phenolic constituents of EVOO are the basis for the beneficial effects of Mediterranean-like diets [5,6,7]. Among these phenolic compounds in EVOO, phenolic alcohols and their secoiridoid derivatives have shown to possess antioxidant, anti-inflammatory, and anti-proliferative bioactivities that are responsible for their properties onhuman health [5,8,9,10]. Oleuropein is the major phenolic compound in olive leaves and drupes. However, during EVOO extraction, oleuropein is hydrolyzed by endogenous glycosidases that drastically reduce its concentration so that only some of its bioactive compounds, in particular the oleacein derivative (dialdehydic form of decarboxymethylelenolic acid linked to hydroxytyrosol; 3,4-DHPEA-EDA) remains as the major secoiridoid derivative in EVOO [11].

In contrast with the high number of studies on the positive impact of oleuropein on health, and its biological characterization, knowledge of the molecular properties of oleacein is still limited. Few available findings refer to the antioxidant and anti-inflammatory activities of this molecule in various experimental models [12,13,14]. Recently, we have provided evidence on the protective action of oleacein on the metabolic alterations that occur in high-fat diet (HFD)-fed mice, demonstrating its preventive effect on body weight gain, obesity-related insulin resistance, and steatohepatitis [15]. To date, however, the molecular mechanism(s) underlying the beneficial effects of oleacein on adipogenesis and peripheral insulin resistance have not been explored intensely, so that its role on insulin sensitivity and its implications for metabolic functions need to be addressed. In the present study, we have evaluated the effects of oleacein-based treatment on the expression of certain markers of adipogenesis and insulin-resistance in vitro in 3T3-L1 adipocytes, and in vivo in adipose tissues from C57BL/6JOlaHsd mice, a strain of mice genetically predisposed to develop obesity when fed with a HFD.

## 2. Materials and Methods

### 2.1. Animal Care and Experimental Protocol

Male C57BL/6JOlaHsd mice were purchased from Envigo RMS (Udine, Italy) and maintained in the animal house facility at “Magna Græcia” University, as reported previously [15]. Two set of experiments were performed. In the first set, 8 five-week-old mice were fed with a normocaloric diet (NCD) (Envigo RMS, TD.2018), 12 were fed with a high fat diet (HFD) (Envigo RMS, TD.06414) and 4 received a HFD with a daily oral gavage of oleacein, 20 mg/kg (HFD-OLEAC) for 5 weeks. In the second set of experiments, 8 mice from the initial HFD group were divided into two new subgroups and continued receiving the HFD either without (HFD group, *n* = 4) or with 20 mg/kg oleacein (HFD-OLEAC group, *n* = 4) for an additional 8 weeks [15]. At the end of both experimental periods, mice were sacrificed, abdominal fat tissues were dissected out, weighed, and stored in liquid nitrogen, or immediately fixed in 10% neutral buffered formalin (Sigma-Aldrich, Milan, Italy) [15]. All experiments were performed in accordance with the guidelines of the Italian (D.M. 116/92) and ECC regulations (O.J. of E.C.L. 358/1 12/18/1986). The experimental protocols were reviewed and approved by the local Ethic Committee of the University “Magna Græcia” of Catanzaro. Precautions were taken to minimize both stress and the number of animals used in each series of experiments.

### 2.2. Histological Analysis

Abdominal fat tissue was dissected from mice and embedded in paraffin with an automated tissue processor (Leica ASP 6025). Serial sections (4 µm thickness) from each paraffin block were mounted on coated glass slides, heated at 60 °C for 60 min, and stained with hematoxylin and eosin for light microscopy analysis. Ten random microscopic areas from independent animals were chosen to measure adipocyte size. Immunohistochemistry was performed using an automated immunostainer (Bond RX; Leica Biosystems, Melbourne, Australia) with antibodies against Glut-4, CD 68, and CD 45 (Novocastra, Leica Microsystems, Milano, Italia), in order to detect macrophages and lymphocytes in the adipose tissue. A direct count of positive cells was conducted by using the cell count function in Image J 1.42 [16] software, on ten fields for all the cases involved in this study. Each field consisted of a photo obtained at 200× or 400× magnification. Glut-4 expression was evaluated by analyzing the intensity of brown signal and applying the following intensity score: 0, absence of brown staining; 1, weak brown staining; 2, moderate brown staining; 3, strong brown staining. Ten fields of sections from all the components of each group were examined blindly by two pathologists, who expressed concordant opinions.

### 2.3. Cell Culture, Differentiation and Oil Red O Staining

3T3-L1 mouse preadipocytes (American Type Culture Collection, Manassas, VA, USA) were cultured in DMEM medium (Thermo Fisher Scientific, Inc., Waltham, MA, USA) supplemented with 10% fetal bovine serum, 100 U/mL penicillin, and 100 µg/mL streptomycin, at 37 °C, in a humidified 5% CO_2_ atmosphere. Cells were induced to differentiate as reported previously [17,18]. In brief, to convert 3T3-L1 cells from fibroblasts to adipocytes, after growth arrest, cells were treated with 500 µmol/L of 3-isobutyl-1-methylxanhine, 1 µmol/L of dexamethasone, and 1 µg/mL of insulin. After four days, the cells were incubated for an additional two days with DMEM containing 1 µg/mLof insulin. The medium was replaced every two days in the presence or absence of oleacein (10 and 100 µM). On day 8, preadipocytes became mature and incubation continued for an additional four days. Lipid droplet accumulation in fully differentiated 3T3-L1 cells was tested by red oil O staining, as described previously [19]. On day 8 and day 12, 3T3-L1 cells were washed and fixed in 10% formaldehyde for 1 h at room temperature. Then, the cells were stained with Oil red O (Sigma-Aldrich, St. Louis, MO, USA) for 20 min and with Mayer’s Hematoxylin (Sigma-Aldrich) for 1 min, and the lipid droplets were observed by a digital transmitted-light inverted imaging system, EVOS XL Core Imaging System (Thermo Fisher Scientific,).

### 2.4. Protein Extraction, Western Blot Analysis and RT-PCR

Total protein extracts were obtained from abdominal fat tissue samples after homogenization in extraction buffer, as previously described [20]. Proteins from 3T3-L1 adipocytes were extracted as reported previously, and protein concentrations were measured by the Bradford assay (Bio-Rad Laboratories, Milan, Italy) [21]. Protein samples (20 µg/lane) were run on a 7%, 9%, or 15% SDS-PAGE gel and then transferred to a polyvinylidene difluoride (PVDF) membranes (VWR, Milan, Italy). The membranes were blocked for 1 h at room temperature (PBS, 0.1% Triton x-100, 5% nonfat dry milk) and incubated with primary antibodies against fatty acid synthase (FAS) (Cell signaling, Danvers, MA, USA), sterol regulatory element-binding transcription factor-1 (SREBP-1), peroxisome proliferator-activated receptor gamma (PPARγ) (Santa Cruz, Heidelberg, Germany), and Adiponectin (Abcam Cambridge, UK) overnight at 4 °C. After washing, the membranes were incubated with horseradish peroxidase (HRP)-conjugated secondary antibodies (Thermo Fisher Scientific), and proteins were visualized with enhanced chemiluminescence (ECL) Plus Reagent (Perkin Elmer, Monza, Italy). Western blot analysis of Glut-4 was performed on skeletal muscle plasma membranes as previously described [2]. qRT-PCR reactions were carried out in triplicates as reported [20,22]. FSP1 mRNA was measured in fat from oleacein-treated and untreated mice, using the following pair of PCR primers: 5′-AGCTACTGACCAGGGAGCTG-3′; 5′-TGCAGGACAGGAAGACACAG-3′.

### 2.5. Statistical Analysis

Results are expressed as mean ± SD. Statistical significance was determined using the one-way analysis of variance, followed by the Tukey multiple comparison test; *p* values < 0.05 were considered statistically significant. All statistical analyses were performed using GraphPad Prism version 5.0 statistical software (GraphPad Software Inc., San Diego, CA, USA).

## 3. Results

### 3.1. Effects of Oleacein on Adipose Tissue In Vivo

**Figure 1:**
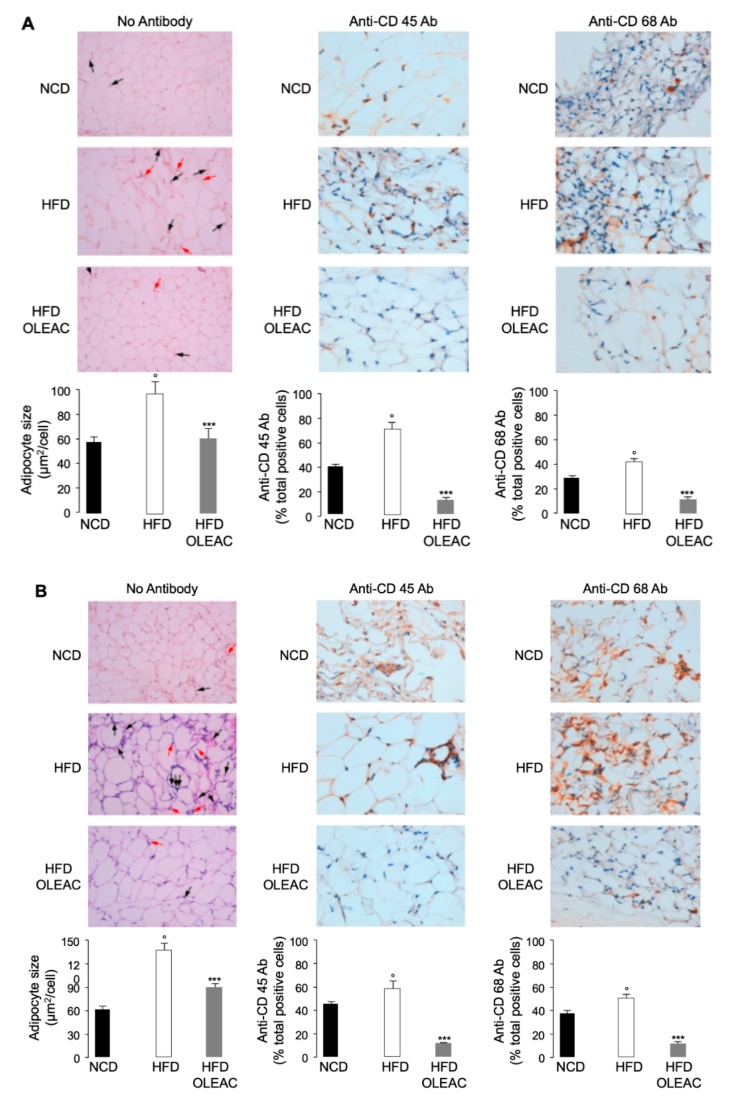
Effects of oleacein on mice adipocytes. Representative color images of hematoxylin and eosin stained fat cells and immunostaining for CD 45 and CD 68 in the abdominal fat tissue of normocaloric diet (NCD) mice and high-fat diet (HFD)-fed mice of the first (**A**) and second (**B**) set of experiments, either untreated (HFD) or treated with oleacein (HFD-OLEAC). NCD, mice under normal caloric diet. Black and red arrows indicate the accumulation of lymphocytes and macrophages within the fat tissue, respectively (200× magnification). Adipocyte diameter and the percentage of total positive cells for CD 45 and CD 68 in abdominal fat tissue are shown in bar graphs. Values are expressed as means ± SD. *** *p* < 0.001 vs. HFD; ° *p* < 0.01 vs. NCD.

The efficacy of oleacein in preventing body weight gain has been demonstrated before by us in HFD-fed mice, showing that treatment with oleacein led to a significant reduction of both liver enlargement and abdominal fat increase [15]. Herein, in the present study, we first performed histological examination of the abdominal fat tissue in HFD-fed mice treated or not with oleacein, and in mice fed with a normal diet. As shown in Figure 1A, after five weeks’ treatment (first set of experiments), oleacein (20 mg/kg) prevented the increase of adipocyte size of HFD-oleacein treated mice, and reduced the inflammatory infiltration of both macrophages and lymphocytes, as assessed by immunohistochemical detection of CD 68 and CD 45 markers, respectively. In line with these findings, mRNA expression of fibroblast-specific protein 1, FSP1, a marker of fibrosis, was lower (about 50% less, *p* < 0.01) in HFD-oleacein treated mice as compared with HFD mice. Similar results on adipocyte cell size and inflammatory infiltrate were observed in a second set of experiments, in which obese mice were treated for an additional 8 weeks with HFD and oleacein at the same dosage regimen (Figure 1B).

### 3.2. Oleacein Regulates Markers of Adipogenesis In Vivo

**Figure 2:**
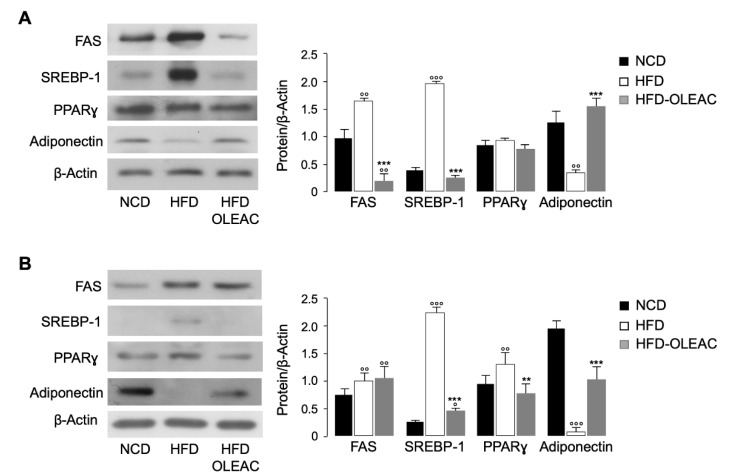
Effects of oleacein on adipogenesis-related markers. Representative Western blots of abdominal fat protein from mice of the first (**A**) and second (**B**) set of experiments. β-Actin, control of protein loading. Densitometric analysis of proteins on Western blots is shown in bar graphs, in each condition. Values are expressed as means ± SD from three experiments performed with four mice from each group. ° *p* < 0.05, °° *p* < 0.01, °°° *p* < 0.001 vs. NCD; ** *p* < 0.01, *** *p* < 0.001 vs. HFD. NCD, normal caloric diet; HFD, high fat diet; HFD-OLEAC, high fat diet-oleacein.FAS, fatty acid synthase.; SREBP-1, sterol regulatory element-binding transcription factor-1, PPARγ, peroxisome proliferator-activated receptor gamma.

Next, we evaluated the expression of some known markers of adipogenesis by performing Western blot analysis on the total protein extracts of adipose tissue from the different groups of mice. In particular, in mice of the first set of experiments, treated with oleacein (HFD-OLEAC), the expression levels of FAS and SREBP-1, two lipogenic factors [23], were reduced compared to HFD-fed mice, whereas the expression levels of Adiponectin were increased in HFD-OLEAC mice (Figure 2A). Overall, these proteins showed similar expression levels (for FAS, even lower) as those in mice fed with NCD (Figure 2A). The expression levels of PPARγ, a transcription factor that regulates genes involved in energy metabolism [24], were unchanged between HFD-OLEAC mice and the HFD mice group (Figure 2A).

In mice that were already obese, treatment with HFD and oleacein (HFD-OLEAC) led to the reduction of PPARγ and SREBP-1 protein expression and the increase of Adiponectin production when compared with the HFD group. No changes were observed in FAS expression under this condition (Figure 2B), indicating that the reduction in adipocyte size observed in HFD-OLEAC mice, which were already obese, could be due to downregulation by oleacein of other key adipose genes involved in adipogenesis and lipid accumulation (e.g., SREBP-1). 

### 3.3. Effects of Oleacein on Glut-4 Expression In Vivo

**Figure 3:**
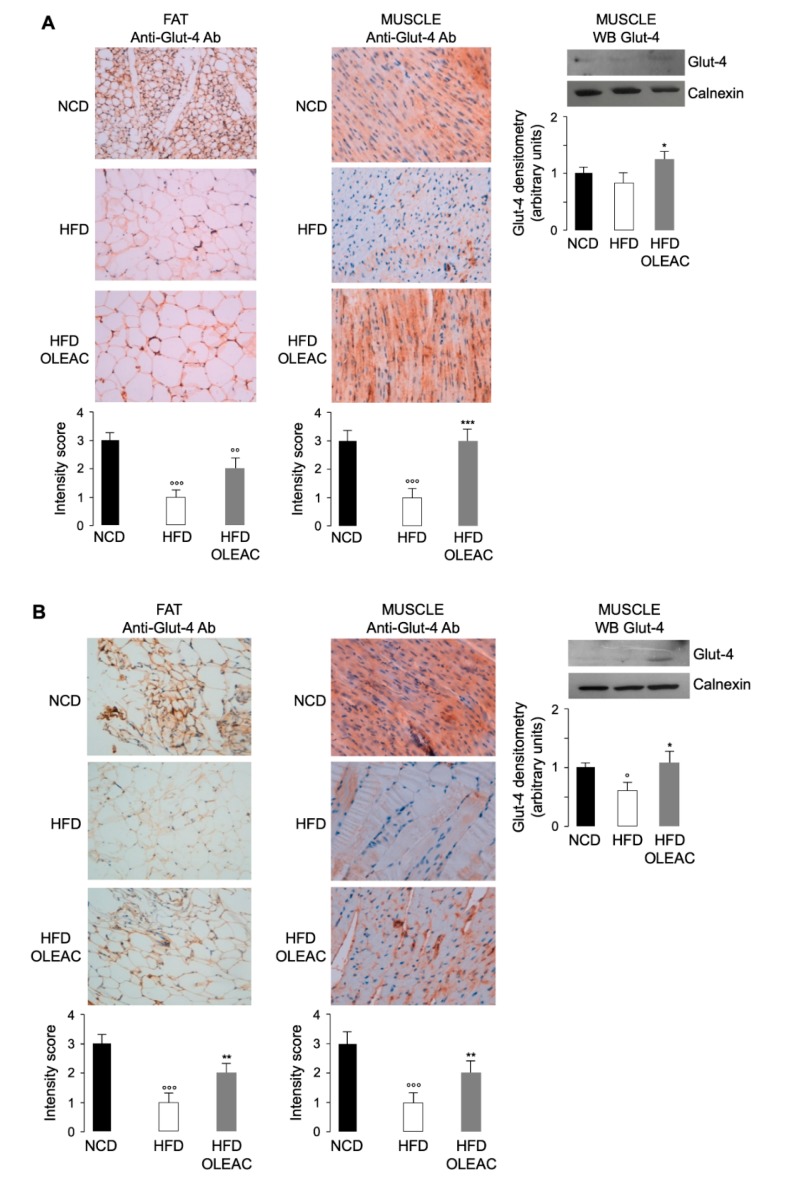
Effects of oleacein on Glut-4 expression. Representative images of immunostaining for Glut-4 in the abdominal fat tissue (200× magnification) and muscle tissue (400× magnification) of NCD and HFD-fed mice of the first (**A**) and second (**B**) set of experiments, either untreated (HFD) or treated with oleacein (HFD-OLEAC). ** *p* < 0.01, *** *p* < 0.001 vs. HFD. °° *p* < 0.01, °°° *p* < 0.001 vs. NCD. NCD, mice under normal caloric diet. Immunostaining intensity score is shown in bar graphs, in each condition. Expression was evaluated by applying an Intensity score: 0, absence of expression; 1, weak expression; 2, moderate expression; 3, strong expression. A representative Glut-4 western blot performed with plasma membrane extracts is shown in both sets of experiments. Densitometry of three independent blots is provided. Data represent mean ± SD for three mice of each group. * *p* < 0.01 vs. HFD; ° *p* < 0.05 vs. NCD.

Based on our previous observations that oleacein effects are correlated with biochemical parameters of improved insulin sensitivity, we investigated the abundance of Glut-4, one of the essential insulin-dependent glucose transporters, in both adipose and muscle tissues of HFD mice, either untreated or treated with oleacein for 5 and 13 weeks. As shown in Figure 3A, positive immunostaining of Glut-4 was observed in abdominal fat and muscle tissues of mice after 5 weeks of treatment with oleacein. This immunoreactivity was found to be higher in HFD-OLEAC mice, as compared to HFD mice. Similar results were observed in mice that underwent oleacein treatment for a further 8 weeks (Figure 3B). In this latter case, the increase in immunoreactivity of Glut-4 paralleled the enhancement of Glut-4 protein expression in muscle plasma membranes from HFD-oleacein treated mice (Figure 3B)

### 3.4. Effects of Oleacein in 3T3-L1 Cells

**Figure 4:**
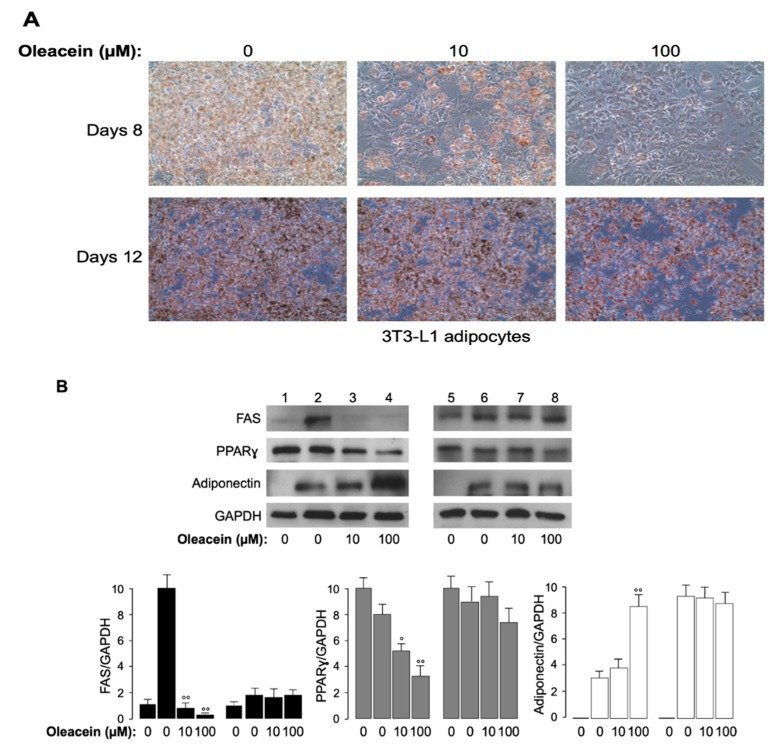
Effects of oleacein on protein expression of adipogenic factors during and after 3T3-L1 adipocyte differentiation. (**A**) Representative images (20×) of cellular lipid droplets in 3T3-L1 treated with increasing concentrations of oleacein during (8 days) and after differentiation (12 days). (**B**) FAS, PPARγ, and Adiponectin were determined by Western blot in oleacein-untreated and treated cells. GAPDH, loading control. Lanes 1 and 5, 3T3-L1 cells before starting differentiation; lanes 2 to 4, 3T3-L1 cells during differentiation; lanes 6 to 8, 3T3-L1 cells after 12 days differentiation. Each picture is a representative of three independent experiments. Densitometric analysis of proteins on Western blots is shown in the bar graph in each condition. Values are expressed as means ± SD from three separate experiments. ° *p* < 0.05, °° *p* < 0.001 vs. oleacein untreated cells (line 2 of Western blots).

Finally, the effects of oleacein were evaluated in vitro, in 3T3-L1 cells, during (8 days) and after (12 days) inducing adipocyte differentiation. 3T3-L1 cells were treated with increasing amounts (0–100 µM) of oleacein over 8 days of cell differentiation and four days thereafter (12 days total). As shown in Figure 4A, the amount of lipid droplets was reduced in oleacein-treated cells, compared with untreated cells, and this reduction was oleacein dose-dependent. Some of the molecular aspects underlying the oleacein-induced anti-adipogenic effects were next investigated by Western blot analyses on protein extracts from treated and oleacein-untreated cells over eight days of cell differentiation and four days thereafter (Figure 4B). Compared with undifferentiated preadipocytes (lanes 1 and 5, Figure 4B), 3T3-L1 cells over 8-days of differentiation (lane 2, Figure 4B) showed the expression of FAS and Adiponectin. During this time period, FAS and PPARγwere reduced in cells treated with both 10 and 100 µM oleacein, whereas the production of Adiponectin was increased (lanes 2 to 4, Figure 4B). In contrast, when oleacein was added to the cells for an additional four days, no effects of oleacein were observed on the expression of these adipogenic markers (lanes 6 to 8, Figure 4B).

## 4. Discussion

A large amount of studies on oleuropein and some of its derivatives have revealed a wide spectrum of positive biological effects for these compounds, including protection against damages caused by HFD [5,6]. In particular, in regard to this latter point, a protective effect of oleuropein against body weight gain and hepatic steatosis has been reported previously by us and others in HFD-induced obesity in animal models [25,26]. Other studies in this context have reported that in vivo administration of oleuropein was able to markedly decrease HFD-induced lipid accumulation [27,28] and inhibit the expression of adipogenesis-stimulating factors during adipocyte differentiation [6,27,29]. However, as mentioned above in the introduction section, the amount of oleuropein in EVOO is very low, making it difficult to justify the “healthy” benefits of this diet component in humans. In contrast, the oleuropein derivative oleacein is one of the most abundant components of EVOO [11], and an efficient sustainable semi-synthesis of oleacein to use as an additive for edible oils has been obtained in our laboratory [30]. Therefore, in the current study, we expanded our previous investigation on the anti-adipogenic action of oleacein in a strain of mice genetically prone to develop obesity under HFD, in which oleacein exhibited strong protective effects against weight gain and liver steatosis, and ameliorated insulin sensitivity and energy homeostasis [15].

Our findings in the present work refer to the effect of oleacein in adipose tissue. By using in vitro and in vivo approaches, we found that protection by oleacein against HFD-induced adiposity (and the co-existent extensive inflammatory infiltration of fat) was mediated through the modulation of major regulators of adipogenesis and was associated with a protective action against insulin resistance. Indeed, histological analysis of adipose tissue from oleacein-treated mice showed that, together with a reduction in adipocyte size, treatment with oleacein attenuated the infiltration of inflammatory cells in adipose tissue, confirming previous results on the anti-inflammatory activity of oleacein [13,14] and its role in preventing both adipose tissue dysfunction and chronic low-grade inflammation, two important factors linking obesity to metabolic disorders. These findings are further supported by the effect of oleacein in preventing the increase of extracellular matrix components. Our investigation of the molecular targets of oleacein in adipose tissue revealed that oleacein may act as a modulator of the expression of some molecular regulators of adipogenesis, such as FAS and SREBP-1, and this is similar with the previously reported effects of oleuropein on adipocyte differentiation [31,32]. Interestingly, in our experimental model, the expression of FAS in oleacein-treated mice was unchanged in overweight/obese animals under HFD. Although this is apparently in contrast with adipocyte size in oleacein-treated and untreated HFD mice, this is in accordance with the previously reported unaltered effect of oleacein on abdominal fat weight [15]. An explanation for this behavior can be related to the fact that, once obesity is established, other molecular mechanisms may take place, differentially influencing the nutraceutical-based treatments. Moreover, our data further underline the importance of PPARγ as a master regulator of adipogenesis, which is also targeted by oleacein. Also, this is similar to what was reported before for oleuropein and various other polyphenols in vitro and in vivo [30,31]. In our model, PPARγ appears to respond to oleacein only following a longer period of treatment, and not in the early phase of weight increase, indicating that the involvement of the PPARγ pathway may occur during the progression of weight gain. 

Another relevant finding of the present work is the effect of oleacein on the expression of Adiponectin, an adipocyte-derived hormone with insulin-sensitizing activity, which is commonly downregulated in obesity and insulin resistance [33]. In all HFD oleacein-treated mice, the expression of Adiponectin was partially or fully restored, further supporting the beneficial role of oleacein in adiposity and adipose tissue dysfunction.

Glut-4 is the major insulin-dependent glucose transporter in muscle and fat, and defects in Glut-4 expression or translocation, at this level, represent a hallmark of peripheral insulin resistance and a harbinger of type 2 diabetes [33,34]. Our previous findings have demonstrated the ability of oleacein to counteract some biochemical parameters of insulin sensitivity. A mechanistic explanation is now offered by the present data on the expression levels of Glut-4, which appears to be a target of oleacein action in adipose and muscle tissues. As demonstrated in our work, Glut-4 is increased at the skeletal muscle plasma membrane level of mice treated with oleacein, indicating a direct role of oleacein on insulin signaling. These metabolic effects of oleacein are further supported by our in vitro studies in 3T3-L1 cells, indicating that, during and after adipocyte cell differentiation, oleacein attenuates intracellular lipid accumulation and reduces the levels of PPARγ and FAS expression, while increasing protein expression of Adiponectin, thereby indicating a beneficial role of oleacein on glucose and lipid metabolism.

In conclusion, these data indicate that oleacein may directly modulate the expression of important regulators of adipogenesis, thereby preventing HFD-induced adiposity and obesity-related insulin resistance. Further studies will clarify these issues and the potential of this nutraceutical as an additive for preventing and treating obesity in humans.
